# Post-partum septic arthritis of the knee: a case report

**DOI:** 10.4076/1757-1626-2-7132

**Published:** 2009-06-10

**Authors:** Shelain Patel, Ravi Trehan, Christian Kinmont

**Affiliations:** 1 Department of Orthopaedics, Kingston HospitalGalsworthy Road, Kingston-upon-ThamesSurrey, KT2 7QB, UK; 2 Department of Orthopaedics, Mayday Hospital530 London RoadCroydon, CR7 7YE, UK

## Abstract

**Introduction:**

Septic arthritis is rare in the post-partum period. This is the first case of a post-partum staphylococcal septic arthritis of the knee reported.

**Case Presentation:**

This report describes a lady who developed symptoms of septic arthritis of the knee within one month of giving birth.

**Conclusion:**

The management of septic arthritis does not differ from standard practice when encountered in the post-partum period. Urgent washout of the joint and antibiotic usage is associated with a favourable outcome.

## Introduction

Joint pain in the lower limbs during pregnancy and in the post-partum period is common [1]. The majority of these cases are due to the increased load of the fetus and placenta, and the alteration in ligament laxity that occurs as a consequence of hormonal changes. Septic arthritis presents with pain in the knee joint, and can be accompanied by a joint effusion, warmth and erythema over the joint, and pyrexia. Septic arthritis of the knee is not common in the post-partum period and only one case has been described previously in the literature, which was Mycoplasma hominis related [2]. Herein we present a case of 25 year old lady who developed a staphylococcal septic arthritis of the knee one month after giving birth.

## Case presentation

A 25-year-old lady presented to hospital 36 days after giving birth via an uncomplicated lower segment caesarean section. She stated that the scar was well healed since the fifth day post-surgery and she had begun breast feeding. There was no history of nipple trauma or mastitis. She complained of a 15 day history of progressive pain, swelling and decreased range of motion in her right knee. Systemic examination revealed pyrexia of 38.5 degrees Celsius and a tachycardia of 120 beats per minute. Examination of the knee demonstrated a red, hot, swollen, tender joint with minimal range of motion. Radiographs of the knee were unremarkable. The knee was aspirated under aseptic conditions and 35 mls of turbid fluid was collected.

Blood laboratory results were as follows: white cell count 15.8 × 10^9^/L, CRP > 250 and ESR 75. Initial gram stain of the aspirate showed 3+ of white cells but no organisms.

In view of the clinical picture, the patient underwent urgent arthroscopic washout of the knee. It was noted that the synovium was thickened and inflamed, and biopsies were taken. Furthermore, there was a significant amount of pus in the suprapatellar pouch. The knee was washed out with nine litres of normal saline.

Cultures of the pus taken from the knee subsequently grew *Staphylococcus aureus* sensitive to flucloxacillin. Cytology of the fluid was negative for crystals. The biopsies taken revealed acute synovitis with extensive ulceration of the synovial lining lined by inflammatory exudate composed of fibrin, polymorphs and scattered macrophages.

The patient was given intravenous benzylpenicillin (1.2 g QDS) and flucloxacillin (1 g QDS) post-operatively and the patient clinically improved. She underwent a further arthroscopic washout of the knee three days after the original procedure. It was found at the second operation that there was some pus in the lateral arthroscopy portal wound though no pus within the knee joint itself.

In total, the patient completed a week's course of intravenous antibiotics and four further weeks of oral antibiotics (Penicillin V 500 mg QDS, Flucloxacillin 500 mg QDS). She was seen in the out-patient clinic 11 weeks after her presentation where she was noted to be doing well with an improved range of motion in her knee of 0-80 degrees.

## Discussion

Acute septic arthritis most commonly develops as a result of haematogenous seeding [3], but may also result from direct introduction [4] or extension from a local focus of infection [5]. It is associated with a high rate of morbidity and mortality if left untreated. The morbidity arises due to the destruction of the articular cartilage with resultant impaired mobility of the joint. Progression to a more severe case is dependent upon the organism involved and a host's own defences [6].

Septic arthritis is uncommon within the post partum period. This may be due to the fact that the post-partum period is oriented towards heightened and activated innate and specific immune defences. Breastfeeding, which the patient was performing, is associated with a boost in these defences [7].

The only previously reported case of post-partum septic arthritis of the knee involved Mycoplasma hominis [2]. This is a commensal bacterium of the vaginal tract. The haematogenous spread of the bacteria in that case was thought to be related to a manual evacuation of the retained products of conception. The most common causative organism overall for septic arthritis is *Staphylococcus aureus* [8] as it was in this case.

We propose that the spread into the joint was haematogenous, with two potential sources. The first is from nipple trauma as a consequence of breastfeeding. Staphylococcal breast abscesses and aseptic reactive arthritis, secondary to staphylococcal breast abscesses, have been described before [9], and thus it is conceivable that this is one potential source. The second potential source is from the surgery of the lower segment caesarean section. Although the delay between surgery and the onset of symptoms was 22 days, the heightened immune response of the postpartum period previously described may have contributed to a long latent phase of infection. This is supported by the fact that the onset of symptoms to presentation at hospital was 15 days which is significantly longer than the average of three days [8].

## Conclusions

This patient presented with clinical and laboratory findings compatible with septic arthritis. Though rare in the post-partum phase, the management does not differ from septic arthritis encountered in patients who are not post-partum. Urgent washout of the joint and use of antibiotics is associated with a favourable outcome.
